# Gauging triple stores with actual biological data

**DOI:** 10.1186/1471-2105-13-S1-S3

**Published:** 2012-01-25

**Authors:** Vladimir Mironov, Nirmala Seethappan, Ward Blondé, Erick Antezana, Andrea Splendiani, Martin Kuiper

**Affiliations:** 1 Dept. Biology, Norwegian University for Science and Technology (NTNU), Trondheim, 7491 Norway; 2 High Performance Computing, Norwegian University for Science and Technology (NTNU), Trondheim, 7491 Norway; 3 Dept. Applied Mathematics, Biometrics and Process Control, Ghent University, Ghent, 9000 Belgium; 4 Dept. Biomathematics and Bioinformatics, Rothamsted Research, Harpenden, AL5 2JQ, UK

## Abstract

**Background:**

Semantic Web technologies have been developed to overcome the limitations of the current Web and conventional data integration solutions. The Semantic Web is expected to link all the data present on the Internet instead of linking just documents. One of the foundations of the Semantic Web technologies is the knowledge representation language Resource Description Framework (RDF). Knowledge expressed in RDF is typically stored in so-called triple stores (also known as RDF stores), from which it can be retrieved with SPARQL, a language designed for querying RDF-based models. The Semantic Web technologies should allow federated queries over multiple triple stores. In this paper we compare the efficiency of a set of biologically relevant queries as applied to a number of different triple store implementations.

**Results:**

Previously we developed a library of queries to guide the use of our knowledge base Cell Cycle Ontology implemented as a triple store. We have now compared the performance of these queries on five non-commercial triple stores: OpenLink Virtuoso (Open-Source Edition), Jena SDB, Jena TDB, SwiftOWLIM and 4Store. We examined three performance aspects: the data uploading time, the query execution time and the scalability. The queries we had chosen addressed diverse ontological or biological questions, and we found that individual store performance was quite query-specific. We identified three groups of queries displaying similar behaviour across the different stores: 1) relatively short response time queries, 2) moderate response time queries and 3) relatively long response time queries. SwiftOWLIM proved to be a winner in the first group, 4Store in the second one and Virtuoso in the third one.

**Conclusions:**

Our analysis showed that some queries behaved idiosyncratically, in a triple store specific manner, mainly with SwiftOWLIM and 4Store. Virtuoso, as expected, displayed a very balanced performance - its load time and its response time for all the tested queries were better than average among the selected stores; it showed a very good scalability and a reasonable run-to-run reproducibility. Jena SDB and Jena TDB were consistently slower than the other three implementations. Our analysis demonstrated that most queries developed for Virtuoso could be successfully used for other implementations.

## Background

Semantic Web (SW) technologies [1,2] are increasingly being adopted by the scientific community, and Life Sciences researchers are no exception [3,4]. Our own contribution to this emerging field has been the development of two semantically integrated knowledge bases - the Cell Cycle Ontology (CCO) and BioGateway [5,6]. SW technologies open a new dimension to data integration, one of the main current challenges in biological knowledge management [4,7,8]. These technologies provide a sound framework to share and combine information via standard data exchange formats. This enables the categorization of information (using ontologies [9]) and fosters the scalability of the integrated system (adaptability to data growth). Moreover, SW technologies provide a sophisticated means to interrogate the originally integrated facts as well as the "hidden" bits of information (via automated reasoning tools [10]). An increasing number of principal biological data providers, such as UniProt [11], have started to make their data available in the SW formats, first of all in the RDF language [12]. In RDF the information is represented in the form of triples subject-predicate-object, *e.g. *"BMC Bioinformatics -has_type- journal". Unlike the current Web, which utilizes just a single semantically undefined predicate (the hyper-link), SW makes use of multiple predicate types with clearly defined semantics.

Data in RDF format is typically stored in dedicated database management systems called triple stores (also known as RDF stores) which expose so-called SPARQL endpoints. Those endpoints allow querying of the store through SPARQL [13], a standard query language recommended by W3C. The triples present in disparate stores form a unified cloud of triples which is accessible for querying from any individual endpoint. A paradigmatic example of such cloud of triples is constituted by the project Linked Open Data [14].

### Triple stores

Currently, there are a number of solutions [15] to store information as RDF triples and they are increasingly becoming adopted by the biological community for the purpose of overcoming some of the limitations (see above) of classical storage solutions (mainly based on relational database management systems).

The development of triple stores has flourished during the last 5 years. Currently, there are more than 20 systems available [16]. Both the academic and private sectors have been involved in developing these triple stores. This race has created a healthy competition leading to rapid progress in key aspects of database management system performance -- querying and loading efficiency, scalability, and stability. The SW community has contributed to this progress by promoting open contests and providing proof-cases for SW applications [17]. It is encouraging for the scientific community that many of these triple stores are freely available for academic use. Obviously, the performance of triple stores is an important issue, especially the response time experienced with SPARQL. The time that a user needs to wait before an answer is returned is a simple but crucial metric that determines the acceptance by the prospective users of the knowledge base in question and the SW technology as a whole. As the number of available triple store implementations is steadily growing, it becomes increasingly difficult to decide which one to use. This necessitates a systematic comparison of the available triple store implementations with respect to their performance (known as benchmarking).

### Benchmarking efforts

Much of the benchmarking done previously on triple stores was based on computationally generated sets of triples that could at best only mimic an actual domain specific knowledge base. Among the "standard" sets used are: the Lehigh University Benchmark (LUBM [18]) and the Berlin SPARQL Benchmark (BSBM [19]), which respectively emulate an organization and e-commerce knowledge bases. In the life sciences domain, studies performed by UniProt [11], demonstrated the current limitations of some triple stores [20]. In this paper we present the 'NTNU SPARQL benchmark', which is based on a Life Science use case, and we report the outcome of the benchmarking of five popular triple store implementations. We tested two additional stores (SwiftOWLIM and 4Store) not included in the previous benchmark experiments [21] and instead of (artificial) computationally generated data, we used biologically relevant real life data from our knowledge base CCO [5], as well as queries which evolved from the direct interaction with the user community.

Our benchmark fills the need for an empirical testing of the performance of triple stores with respect to queries and data whose characteristics are representative of Life Sciences information.

## Results

### The NTNU dataset

The dataset used in our analysis consists of ten RDF graphs constituting the Cell Cycle Ontology (CCO) [5] (a total of 11.315.866 triples). There are four taxon-specific graphs (for *H. sapiens, A. thaliana, S. cerevisiae, S. pombe*) and an integrated graph. All of them share a core set of triples (ontologies) and a set of taxon-specific triples. The integrated graph contains additional orthology relations for proteins. Each of the five graphs has a counterpart graph augmented with pre-computed transitive and reflexive closures.

More details on the characteristics of this dataset can be found in Table 1.

**Table 1:** Characteristics of the NTNU dataset.

Graph	# triples	# classes	Max # sup	Avg # sup	# relations	# relation types
cco	2503040	89526	33	7.72	461946	30
cco_tc	3170556	89526	33	7.72	1129462	30
cco_A_thaliana	356903	12578	34	9.11	22132	30
cco_A_thaliana_tc	469484	12578	34	9.11	134713	30
cco_S_cerevisae	842344	35004	34	7.99	171825	30
cco_S_cerevisae_tc	1120545	35004	34	7.99	450026	30
cco_S_pombe	406131	14584	34	8.86	39997	30
cco_S_pombe_tc	533481	14584	34	8.86	167347	30
cco_H_sapiens	836622	29187	34	8.29	121383	30
cco_H_sapience_tc	1076760	29187	34	8.29	361521	30

A list is shown of the characteristics of the 10 graphs constituting the NTNU dataset. Reported in this table are, for each graph: the number of triples, the number of classes (the basic units in CCO), the maximum number of super classes for a class in the graph (Max #sup), the number of super classes averaged over all the classes (Avg #sup), the number of relations (predicates between two classes) and the number of distinct relation types. For technical reasons the analysis of the super class statistics was performed on random selections of 10000 classes.

### The set of queries

In our analysis we used 24 queries which were defined to answer real life questions identified in close collaboration with the end users of CCO (Additional file 1). Our query set encompasses a broad range of SPARQL features and combinations thereof, as summarised in Table 2. As shown in this table, queries used in our analysis ensure a comprehensive assessment of the performance of the triple stores with respect to a real Life Science use case.

**Table 2:** Overview of the query features.

	Simple Filters	More than 8 triple patterns	OPTIONAL operator	LIMIT modifier	ORDER BY modifier	DISTINCT modifier	REGEX operator	UNION operator	COUNT operator
**Q1**						x			
**Q2**								x	
**Q3**	x	x	x			x	x		
**Q4**									
**Q5**									
**Q6**									
**Q7**	x						x		
**Q8**						x			
**Q9**	x								
**Q10**	x	x				x	x		
**Q11**									
**Q12**								x	
**Q13**		x	x			x		x	
**Q14**					x				
**Q15**									
**Q16**									
**Q17**						x			x
**Q18**	x	x			x		x	x	
**Q19**						x		x	x
**Q20**						x			x
**Q21**						x			x
**Q22**									
**Q23**									
**Q24**	x			x			x		

The selected 24 queries (Q1 through Q24) were used to evaluate the triple stores' responsiveness with respect to various query features. The table shows the full set of queries and the features used therein.

### Overall performance

A summary of the results of our analysis on the five selected triple stores is reported in Table 3, which summarize loading times and query answering.

**Table 3:** Overall performance.

Store	Load time	Query time	RSE
Virtuoso	527	204	0.053
Jena SDB	2005	730	0.020
Jena TDB	833	1446	0.235
Owlim	230	14258	0.156
4Stroe	128	47567	0.097
AVG	745	12841	0.112

**Load time **- the total load time in seconds for the 10 graphs averaged over the three replicates. **Query time **- ARTs in seconds summed over all the data points (24 queries and 10 graphs). **RSE **- the relative standard errors averaged over all the data points (24 queries and 10 graphs).

First of all, we compared the loading performance of the five selected stores. Loading performance is of paramount importance in the Life Sciences domain, as primary data sources are frequently updated and the content of triple stores needs to be maintained up-to-date. In this respect 4Store shows an exceptionally good performance, followed by SwiftOWLIM.

In order to get a bird's-eye view on the querying performance of the selected stores, we aggregated the averaged response times (ART, see Methods) into a single cumulative total response time and estimated the average relative standard errors for each of the stores. (Note that the version of SwiftOWLIM used in our experiment did not support the COUNT operator, therefore the values for this store do not include data for queries Q17, Q19, Q20 and Q21). The total query execution time varied in a very broad range over the triple stores (Table 3).

Load times have been found consistent across test-runs, whereas query answering times have presented some significant variation (most notably 4Store and SwiftOWLIM), even though individual test runs were performed in equivalent conditions (see Methods). Variability could be explained by the different environment of the operating system during different runs.

On the basis of these data, Virtuoso emerges as an overall winner, however, the picture changes significantly when we look into the query-specific behaviour.

### Query-specific performance

Table 4 makes clear that all the stores behave in a query-specific manner. A highly query-specific behaviour has also been observed by Bizer and Schultz [22]. Nevertheless, a couple of common trends are discernible. SwiftOWLIM is by far the best performer with the relatively short ART queries; 4Store shows the best performance with the moderate ART queries; and Virtuoso is doing best of all with the long ART queries. Jena SDB is consistently the slowest store with all the short and moderate ART queries. Virtuoso is the only store whose ARTs are better than average for all the 24 queries.

**Table 4:** Query-specific performance.

Query	Virtuoso	Jena SDB	Jena TDB	4Store	OWLIM	Avg
Q5	2.639	13.446	11.000	1.526	0.408	5.804
Q23	5.630	13.343	10.454	1.343	0.009	6.156
Q11	5.343	13.339	10.703	1.419	0.011	6.163
Q16	5.617	13.345	10.825	1.346	0.009	6.228
Q15	6.163	13.342	10.544	1.390	0.018	6.291
Q22	5.170	13.709	10.981	1.428	0.173	6.292
Q4	5.916	13.348	10.773	1.539	0.017	6.319
Q8	7.094	13.336	10.449	1.577	0.049	6.501
Q12	7.198	13.373	10.731	1.400	0.030	6.546
Q2	7.281	13.337	10.768	1.438	0.052	6.575
Q6	4.054	14.523	10.573	1.779	2.020	6.590
Q19	2.065	13.390	9.795	1.326		6.644
Q9	5.820	13.711	10.699	2.133	1.067	6.686
Q21	3.379	13.335	9.818	1.316		6.962
Q10	4.679	13.757	11.676	2.529	4.664	7.461
Q17	5.648	13.390	10.119	1.350		7.627
Q20	6.110	13.387	10.686	1.315		7.875
Q1	1.897	18.064	14.258	1.647	8.024	8.778
Q13	1.658	52.545	14.156	1.569	0.034	13.992
Q24	2.813	24.719	38.619	14.366	27.242	21.552
Q7	2.617	26.519	39.248	14.110	28.996	22.298
Q14	5.775	13.338	46.433	1.401	91.894	31.768
Q3	3.358	30.476	27.049	3.654	1121.702	237.248
Q18	22.840	76.013	493.596	24999.569	8325.734	6783.550

The ARTs in seconds summed over the 10 graphs. The queries are sorted in the order of the response time averaged over the 5 stores (**Avg**).

The three slowest queries, Q14, Q3 and Q18, display extremely idiosyncratic behaviour and defy any generalization. Two of them affect specifically the performance of SwiftOWLIM - Q14 (mildly) and Q3, the only two cases where SwiftOWLIM shows the slowest response of all the stores. The impact of Q18 is more severe and it affects the performance of Jena TDB, SwiftOWLIM and 4Store in the order of magnitude. In the case of 4Store the ART for Q18 is 9141 (sic !) times longer than the ART of this store averaged over all the other 23 queries. Both Q14 and Q18 include the ORDER BY modifier, not used by any other query, which might suggest that the implementation of this feature is suboptimal in the affected stores. However, this proposition is in conflict with the response of 4Store to these two queries (17859 fold difference in the ARTs). On the other hand, Q3 does not make use of any unique features; instead it includes a rather wide range of features found as well in other queries. At present it is not possible to determine whether any particular combination of these features is responsible for the long execution time.

### Scalability

**Figure 1:**
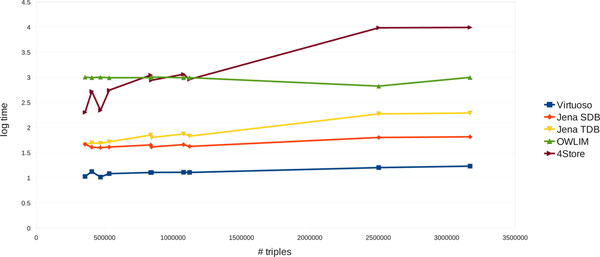
**The scalability of the five stores**. The ARTs were summed over all the 24 queries (except for the queries Q17, Q19, Q20, Q21 for SwiftOWLIM) and plotted against the total number of triples in the graphs (Y axis is logarithmic).

**Figure 2:**
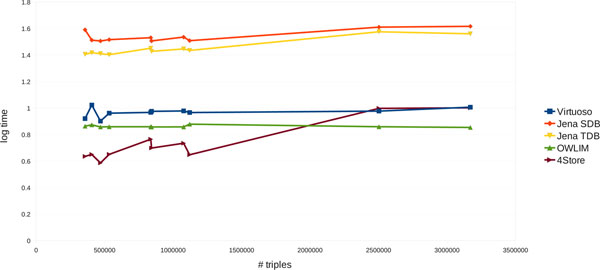
**The scalability of the five stores in the absence of outliers**. Cumulative ARTs are shown, plotted against the total number of triples in the graphs. This figure is similar to Fig. 1 but omitting the queries Q3, Q14 and Q18.

Finally, we wanted to see how well the ARTs scale up with respect to the graph sizes. The ARTs were summed over all the queries (except for the queries Q17, Q19, Q20. Q21 for SwiftOWLIM) and plotted against the total number of triples (Figure 1). The figure shows that SwiftOWLIM scales up exceptionally well, followed by Virtuoso. In contrast, 4Store demonstrated the poorest performance with respect to scalability. However, as pointed out earlier, the behaviour of SwiftOWLIM and 4Store is strongly affected by a few outliers. Therefore, to eliminate the impact of the outliers we excluded the three slowest queries Q3, Q14 and Q18 from the plot (Figure 2). Although the mutual arrangement of the individual graphs on the plot changed in favour of SwiftOWLIM and 4Store, the conclusion about the scalability drawn above did not change. It should be noted that the fact that queries were developed using Virtuoso should not have any impact on the scalability.

## Discussion

Our benchmark is intended to represent a realistic usage of triple stores in the Life Sciences context. It is designed to measure loading and query answering times, which are key parameters in the choice of a triple store. However, there are other considerations to be made when selecting a triple store for a specific use case.

One of these considerations is the architecture for which a triple store is designed, and in particular whether this can be deployed on a cluster-based architecture. Some of the systems we have benchmarked (4Store, Virtuoso) can be deployed on a cluster making an efficient use of this affordable parallel architecture, which promises better scalability for very large datasets. As a note, SwiftOWLIM, which we have tested, is designed for optimal performance for datasets below 100M triples, whereas BigOWLIM is designed for large datasets and offers improved performance on a cluster-based architecture.

Triple stores vary also in the programmatic access they offer and in the features they support. As an example, the big difference in performance between TDB and SDB (which are both from the same software suite) are compensated by the different functionalities they offer. SDB performs less well in queries, but provides transactional support and concurrent access, which are not offered by TDB.

It should also be noted that the query performance of triple stores is dependent on the efficiency of their query optimization engines. The relevance of such optimization depends on the usage conditions of triple stores. Where a few queries are routinely executed, optimization can be performed by users at design time and the lack of an optimizer is not penalizing. TDB is a peculiar case in that it offers an optimizer which is statistics based, and which needs to be updated after a change in the data content.

Query performance may also depend on the characteristics of datasets in ways which can be difficult to detect. For example, we have observed that 4Store loading times scale well with respect to the total number of triples, while the number of different properties in the dataset could be a limiting factor.

It should be noted that as our queries have evolved from real use cases on the CCO ontology, which has been mostly accessed via a Virtuoso-based SPARQL endpoint. It is not possible to rule out that queries displaying idiosyncratic behaviour towards Virtuoso were naturally avoided or optimized. For this reason, we have provided figures which don't take such outliers in consideration. At the same time, it should be noted that neither SDB nor TDB show any particular outlier behaviour even though our queries were not optimised for these stores. We hope that the identification of such idiosyncratic behaviour will anyway be useful for the respective development communities.

Finally, we did not include in our testing any element directly related to reasoning performance, which is a relevant issue in the Life Sciences, where simple chains of transitive properties are common in biomedical terminologies. Testing reasoning features would have introduced an additional layer of complexity in the interpretation of results and we have preferred to focus on testing basic query-answering performance. For this reason, we have simulated transitive properties via a materialization of inferred triples (see section Methods).

## Conclusions

We have compared the performance of a set of biologically relevant queries in five popular triple stores, OpenLink Virtuoso (Open-Source Edition), Jena SDB, Jena TDB, SwiftOWLIM and 4Store. We have used a dataset and a set of queries which are representative of a real life sciences application case. We have focused in our analysis on loading time, query time and its reproducibility, under "common" deployment and usage conditions.

In general, the performance proved to be quite query-specific. Nevertheless, it was possible to identify three groups of queries displaying similar behaviour with respect to the different stores:

queries with relatively short ART,

queries with moderate ART,

queries with relatively long ART.

SwiftOWLIM proved to be a winner in the first group, 4store in the second one and Virtuoso in the third one. Virtuoso emerged from our analysis as a very balanced performer in our application case - its upload time and response time for all the 24 queries were better than average among the tested stores and it showed a very good scalability. Even though in our study we used only moderately large triple stores (~11 M triples), others demonstrated that Virtuoso excels when confronted with much larger stores, up to 100-200 M triples [19,22]. From our experience we conclude that Virtuoso is well suited for managing large volumes of biological data as is the case of our BioGateway project where it gracefully supports querying over ~1.8 billion triples [23].

### Note added in proof

After the completion of this work Berlin Benchmark v.3 has been released. A line-up comparable to the one in our work (Virtuoso, Jena TDB, 4store, BigData, and BigOWLIM) was used for benchmarking and the conclusions reached are similar to ours [24].

## Methods

### Software

The set of triple store implementations included Virtuoso OpenSource 6.0.0, SwiftOWLIM 2.9.1, 4Store 1.0.2, Jena SDB 1.3.1, Jena TDB 0.8.2. The stores were run under CentOS 5.5 operating system.

### Configuration

Default configurations were used except the parameters listed below.

### Virtuoso

FileExtend = 100000

MaxCheckpointRemap = 1048576

ServerThreads = 100

CheckpointInterval = 600

O_DIRECT = 1

NumberOfBuffers = 500000

MaxDirtyBuffers = 18000

MaxStaticCursorRows = 50000

TransactionAfterImageLimit = 50000000

FreeTextBatchSize = 10000000

[Striping]

Segment1 = 1536M, db-seg1-1.db, db-seg1-3.db, db-seg1-2.db

...

Segment32 = 1536M, db-seg32-1.db, db-seg32-3.db, db-seg32-2.db

MaxQueryCostEstimationTime = 84400

MaxQueryExecutionTime = 7200

### Swift Owlim

owlim_control.sh:

$JAVA_HOME/bin/java -Xmx4096m -cp "$CP_TESTS:$EXT_LIBS"

owlim.properties:

num.threads.run: 10

query.language: sparql

preload.format: rdfxmls

owlim.ttl:

owlim:entity-index-size "200000"

owlim:jobsize "200"

### Jena SDB

sdb:layout         "layout2/index"

### Hardware

The analysis was performed on a Dell R900 machine with 24 Intel(R) Xeon(R) CPUs (2.66GHz). The machine was equipped with 132G main memory and 14x500GB 15K SAS hard drives.

### Testing procedure

Outline

for each triplestore {

         repeat 3 times {

                  delete db files

                  switch on triplestore

                  load data

                  run all queries (in exactly the same order)

                  switch off triplestore

         }

}

The ten RDF graphs constituting the CCO [5] were used for the analysis (11.315.866 triples in total, Table 1 for more details).

The graphs were queried with the 24 SPARQL queries from the library of queries on the CCO web site [25], (see Additional File 2). The queries were executed on each of the graphs consecutively from query Q1 through Q24. The experiments were replicated three times. Prior to each experiment the contents of the stores were completely cleared and uploaded anew.

### Data analysis

The average response times and the corresponding standard errors for these three observations were computed for all the data points (24 queries and 10 graphs, available at [23]) and used to aggregate the data for Tables 3 and 4 and Figures 1 and 2. The relative standard errors (RSE) were produced by dividing standard errors by the corresponding ART. Also, the total data load times (10 graphs) were averaged over the three replicates and presented in Table 3.

### Data availability

The RDF files used for uploading the triple stores are downloadable from the CCO web site [26], the queries used in our analysis are avaiable as Additional File 2.

## List of abbreviations

ART: averaged response time; CCO: Cell Cycle Ontology; RDF: Resource Description Framework; RSE: Relative Standard Error; SW: Semantic Web; W3C: World Wide Web Consortium; NTNU: Norwegian University for Science and Technology; LUBM: the Lehigh University Benchmark; BSBM: the Berlin SPARQL Benchmark; SPARQL: SPARQL Protocol and Query Language.

## Competing interests

The authors declare that they have no competing interests.

## Authors' contributions

VM conceived and designed the experiment, oversaw its execution, analysed and interpreted the results and drafted the manuscript; NS carried out the experiment; WB developed the queries and contributed to the drafting of the manuscript; EA and AS contributed to the analysis and interpretation of the test results and to the drafting of the manuscript; MK was responsible for the project, oversaw its execution and oversaw and contributed to the drafting of the manuscript. All authors approved the final version of the paper.

## Supplementary Material

Additional file 1 **benchmarking results**. This file contains the raw and derived data values. There is one sheet with the upload data and one separate sheet for each of the stores with the query response times. Additionally there is a sheet with the aggregated data which were used to generate the tables and figures in the manuscript. Click here for file

Additional file 2 **SPARQL queries**. This file contains the SPARQL code of the 24 queries used for testing the stores. Click here for file
